# Development and Investigation of Hierarchically Structured Thin-Film Nanocomposite Membranes from Polyamide/Chitosan Succinate Embedded with a Metal-Organic Framework (Fe-BTC) for Pervaporation

**DOI:** 10.3390/membranes12100967

**Published:** 2022-10-02

**Authors:** Tatiana Plisko, Katsiaryna Burts, Andrey Zolotarev, Alexandr Bildyukevich, Mariia Dmitrenko, Anna Kuzminova, Sergey Ermakov, Anastasia Penkova

**Affiliations:** 1 Institute of Physical Organic Chemistry, National Academy of Sciences of Belarus, 220072 Minsk, Belarus; 2 St. Petersburg State University, 7/9 Universitetskaya nab., 199034 St. Petersburg, Russia

**Keywords:** chitosan succinate, polyamide, thin film nanocomposite membrane, dynamic technique, interfacial polymerization, interlayer, metal-organic framework, Fe-BTC, pervaporation, isopropanol dehydration

## Abstract

Thin-film composite membranes (TFC) obtained by the formation of a selective layer on a porous membrane-substrate via interfacial polymerization (IP) are indispensable for separation procedures in reverse osmosis, nanofiltration, pervaporation, and gas separation. Achieving high selectivity and permeability for TFC membranes is still one of the main challenges in membrane science and technology. This study focuses on the development of thin film nanocomposite (TFN) membranes with a hierarchically structured polyamide (PA)/chitosan succinate (ChS) selective layer embedded with a metal–organic framework of iron 1,3,5-benzenetricarboxylate (Fe-BTC) for the enhanced pervaporation dehydration of isopropanol. The aim of this work was to study the effect of Fe-BTC incorporation into the ChS interlayer and PA selective layer, obtained via IP, on the structure, properties, and performance of pervaporation TFN membranes. The structure and hydrophilicity of the developed TFN membranes were investigated using scanning electron microscopy (SEM) and atomic force microscopy (AFM), along with water contact angle measurements. The developed TFN membranes were studied in the pervaporation dehydration of isopropanol (12–30 wt % water). It was found that incorporation of Fe-BTC into the ChS interlayer yielded the formation of a smoother, more uniform, and defect-free PA ultrathin selective layer via IP, due to the amorpho-crystalline structure of particles serving as the amine storage reservoir and led to an increase in membrane selectivity toward water, and a slight decrease in permeation flux compared to the ChS interlayered TFC membranes. The best pervaporation performance was demonstrated by the TFN membrane with a ChS-Fe-BTC interlayer and the addition of 0.03 wt % Fe-BTC in the PA layer, yielding a permeation flux of 197–826 g·m^−2^·h^−1^ and 98.50–99.99 wt % water in the permeate, in the pervaporation separation of isopropanol/water mixtures (12–30 wt % water).

## 1. Introduction

Nowadays, approaches to the selective extraction of components from their mixtures using environmentally friendly, low-energy-consuming, and safe technologies are the focus of attention for both researchers and workers in the industry. The membrane separation process via pervaporation features these advantages, along with the lack of a need for additional reagents and the high selectivity and separation efficiency of various water-organic mixtures and mixtures of organic substances, in comparison with other traditional separation processes (distillation, azeotropic and extractive distillation, extraction and adsorption, etc.) [1]. Pervaporation is promising for the separation of organic-organic mixtures, such as benzene/cyclohexane, methanol/methyl tert-butyl ether, and ethanol/ethyl tert-butyl ether, as well as mixtures of isomers [2], and is widely applied for the dehydration of organic solvents (for example alcohols, pyridine, acetic acid, etc.), especially for the separation of their azeotropic mixtures. The rapid development and active use of pervaporation require the development of new and highly efficient membranes.

Thin-film composite membranes (TFC), obtained by the formation of a selective layer on a porous membrane-substrate, are indispensable for separation in such membrane processes as reverse osmosis, nanofiltration, pervaporation, and gas separation [3,4,5,6]. TFC membranes are used in brine and brackish water desalination [7], water treatment [8,9], natural gas and biogas upgrading [10,11], gas separation [12], the dehydration and purification of organic solvents [13,14], and the concentration and purification in the pharmaceutical, food and biotechnology industries [15]. TFC membranes consist of several layers with a certain pore size, size distribution, and thickness. TFC membranes are composed of a non-woven substrate (thickness: ∼100–200 µm and pore size: ∼5 µm), anisotropic porous ultrafiltration membrane-substrate (thickness: ∼50–150 µm, pore size ∼0.01–0.1 µm) and an ultrathin selective layer consisting of a nanofilm with a thickness ranging from a few nanometers to hundreds of nanometers [16]. Depending on the membrane process, an ultrathin selective layer can be porous (nanofiltration) and non-porous (reverse osmosis, gas separation, pervaporation). An ultrathin selective layer provides selective separation and high permeability (due to the thin layer thickness), while the porous membrane support provides mechanical strength and membrane integrity without affecting the mass transfer.

Interfacial polymerization (IP) is the most commonly used method for the formation of ultrathin selective layers with a given structure on the surface of a porous membrane support for various separation processes [17]. This technique is applied for the formation of an ultrathin functional polymer layer at a phase boundary by a reaction between two immiscible solutions, such as an aqueous solution of di- or multifunctional amine and a solution of di- or multifunctional acyl chloride in an organic solvent. The most important advantages of the IP, which determines its wide application in industry, are mild reaction conditions (room temperature and pressure), low sensitivity to reaction conditions, monomer purity and their ratios, an extremely high reaction rate, and the possibility of formation of ultrathin films of an unlimited area [18]. The IP reaction is self-inhibiting, which makes it possible to obtain very thin selective layers and has the property of “self-healing” in the defective regions of an ultrathin film [19]. TFC membranes with a selective layer, based on polyamide obtained by IP, are currently the “gold standard” of membranes for nanofiltration and reverse osmosis [5,7]. IP can also be applied to obtain TFC membranes for pervaporation and gas separation. However, the use of IP is not very widespread for pervaporation and gas separation membranes due to difficulties in controlling the interfacial polymerization process, which impedes the formation of a thin but dense and defect-free layer [20]. The greater part of current research efforts is focused on overcoming the trade-off between the permeability and selectivity of the TFC membranes prepared by IP [17,18]. It has been reported in the literature that pervaporation TFC membranes prepared by IP were studied for the dehydration of alcohols (ethanol [6], isopropanol [21,22,23], and tert-butanol [24]), ethylene glycol [25,26], tetrahydrofuran [27], seawater desalination [28], and the separation of methanol/methyl tert-butyl ether mixtures [29].

The formation of a thin selective layer of pervaporation TFC membranes by IP is controlled by such factors as (1) substrate (porosity, pore size, hydrophilicity, roughness); (2) the nature and concentration of monomers (amine and acyl chloride); (3) the interlayer between the membrane substrate and selective layer formed by IP (gutter layer), its nature and its structure; (4) additives in the selective layer (e.g. nanoparticles, nanomaterials, surfactants, hydrophilic polymers, and multifunctional additives); (4) solvents for monomers; (5) the conditions of the IP process (immersion time and reaction time) [3,4,5,6,13,29,30,31,32,33].

The formation of the selective layer by IP is highly dependent on the properties of the substrate membrane. To obtain TFC membranes, both ultra- and microfiltration membranes can be used as support layers; these are usually prepared using the phase inversion method. As a rule, during interfacial polymerization, the substrate is treated with an aqueous solution of the first monomer (amine) at the first stage, then the solution of the second monomer (acyl chloride), in an organic solvent, is in contact with this substrate. When two immiscible phases come into contact, the amine component diffuses to the interface (the limiting stage of interfacial polymerization). The number of amine molecules participating in the reaction strongly depends on the properties of the substrate membrane (pore size, porosity, hydrophilicity) [13,34,35,36,37,38,39,40,41]. To tune the physicochemical properties of the membrane substrates, different modification strategies are applied: the addition of hydrophilic oligo- or polymers, inorganic particles, hydrogels, etc., into the structure of the substrate [42,43]; reactive surfaces [44,45]; or the formation of a highly permeable and microporous interlayer between the substrate and the polyamide layer [29,30,31,32,33,46].

One of the most efficient approaches to modulate substrate properties is to form an interlayer between the substrate membrane and the polyamide layer, obtained by IP [29,30,31,32,33]. The formation of the interlayer promotes the better interaction of the surface with the monomers used in interfacial polymerization. The interlayer can also accumulate the amine component and, thus, control and facilitate the process of interfacial polymerization via (1) the increased storage of amine; (2) controlled amine diffusion; (3) regulated nuclei formation; (4) interfered heat and nanobubble production; and (5) inhibited downward growth of polyamide [31]. The interlayer usually has small, similarly sized pores, high porosity, and surface hydrophilicity. Such surface properties contribute to the formation of a very thin and defect-free selective layer. However, for gas separation, the TFC membrane interlayer is often formed from a hydrophobic, but highly permeable, polydimethylsiloxane [29,30,31,32,33].

Three main types of interlayers were reported in the literature: organic coatings, nanomaterials, and nanocomposite coatings, which consist of organic coatings embedded with nanomaterials [29,30,31,32,33]. Different materials for interlayer formation for TFC membranes were studied: polymers (polydopamine [47,48] and a mixture with polyethyleneimine [49], polyamide [50], chitosan [51], sulfonated poly (ether ether ketone) [52], polyvinyl alcohol [53,54], and polydimethylsiloxane), TiO_2_ nanoparticles [21], graphene oxide and its derivatives [55,56], carbon nanotubes [57,58], covalent organic frameworks (COF) [59,60,61], tannic acid/Fe^3+^ nanoscaffolds [62], cellulose nanocrystals [63], metal–organic frameworks (MOF) [64,65], polymer nanocomposites (graphene oxide/polydopamine [66], silver nanoparticles /polydopamine [67], halloysite nanotubes/polydopamine [68], and COF/polydopamine [69]), as well as complexes of the polyelectrolyte (poly (sodium 4-styrenesulfonate)) and metal ions (Fe^3 +^ ) [70].

According to the literature review, the studies on the effect of interlayers on the performance of TFC membranes have been focused mainly on nanofiltration and reverse osmosis membranes. Only a few works were reported on the application of an interlayer in the development of TFC membranes via IP for pervaporation: the deposition of the polydopamine interlayer on a polyethersulfone support [26], a cross-linked chitosan layer on a polyacrylionitrile support [51], a TiO_2_ layer on an α-Al_2_O_3_ hollow fiber support [51], a polyamide layer via IP on a nanofibrous substrate [50], and modified carbon nanotubes on a hydrolyzed polyacrylonitrile substrate [71]. In all cases, permeation flux and membrane selectivity were improved or, sometimes, selectivity was maintained at the same high level, due to the construction of an interlayer [26,50,51,71].

Achieving the desired selectivity and permeability for TFC is still one of the main challenges in the fabrication of TFC membranes [72]. One of the most common approaches to tackling this challenge is the use of additives during the synthesis of a selective layer by IP. For this purpose, surfactants, nanoparticles and nanomaterials, hydrophilic polymers, and multifunctional additives are added to either an aqueous or organic phase during IP [73,74,75,76]. More often than not, nanomaterials are used as modifying agents that can improve the characteristics of the polyamide layer: increasing hydrophilicity, anti-fouling, catalytic ability, narrow dispersed nanochannels, and so on. One of the key roles of nanomaterial additives is creating additional mass transport pathways. Typical nanomaterials with intrinsic pores/channels include carbon nanotubes [77], titanate nanotubes [78], metal–organic frameworks [79,80], and macrocyclic molecules [81]. More recently, two-dimensional (2D) laminar channels that are formed from 2D nanosheets have drawn increasing attention in the field of selective mass transport [82].

The aim of this work was to study the effect of incorporating a metal–organic framework (Fe-BTC) into a chitosan succinate interlayer and polyamide selective layer, obtained via interfacial polymerization on the structure, properties, and performance of TFN membranes for isopropanol dehydration via pervaporation. The novelty of this work is that succinate chitosan, embedded with Fe-BTC, was used for the first time as an interlayer for interfacial polymerized polyamide membranes for pervaporation. Moreover, the synergistic modification of interlayers and polyamide selective layers with a metal-organic framework (Fe-BTC) is investigated here for the first time in the context of TFC pervaporation membranes. 

## 2. Materials and Methods

### 2.1. Materials

Ultrafiltration polyacrylonitrile (PAN) membranes with a molecular weight cut-off (MWCO) of 100 kDa and pure water flux of 200–245 L·m^−2^·h^−1^ (at a transmembrane pressure of 0.1 MPa) (manufactured by the Institute of Physical Organic Chemistry of the National Academy of Sciences of Belarus, Minsk, Belarus) were used as a porous membrane support for the preparation of thin-film composite (TFC) and thin-film nanocomposite (TFN) membranes. Chitosan succinate (ChS, M~30,000 g·mol^−1^, Bioprogress, Moscow, Russia) was chosen for the formation of an interlayer of TFC membranes. Microparticles of a metal–organic framework (MOF) based on iron 1,3,5-benzenetricarboxylate (Fe-BTC, Basolite® F300, Sigma Aldrich, St. Louis, MO, USA) were used as an additive to the ChS interlayer and polyamide selective layer of TFC and TFN membranes. To disperse the Fe-BTC in aqueous ChS solutions, ethylene-diamine-tetra-acetic acid disodium salt (EDTANa, Sigma Aldrich, St. Louis, MO, USA) was applied. ChS was crosslinked using maleic anhydride (MA, Vekton, St. Petersburg, Russia). Triethylenetetramine (TETA, FINSAD Group, Helsinki, Finland) and trimesoyl chloride (TMC, Sigma Aldrich, St. Louis, MO, USA) were used as monomers for the formation of a polyamide selective layer by interfacial polymerization. Distilled water was used as a solvent for the TETA solution preparation. Hexane (Belhim, Minsk, Belarus) served as a solvent for the TMC. All materials were used without prior purification.

### 2.2. Preparation of TFC Membranes

#### 2.2.1. Formation of the ChS and ChS/Fe-BTC Interlayer

The preparation of ChS/MA aqueous solutions, as well as the formation of a ChS interlayer on the PAN membrane-support in the dynamic mode, were reported in our previous work [83]. Briefly, a ChS interlayer on the surface of the porous PAN membrane support was formed using an aqueous solution containing 1.0 wt % ChS and 0.15 wt % maleic anhydride (MA). The solution for the formation of a ChS interlayer embedded with Fe-BTC was prepared in the following way: 2 wt % of an aqueous dispersion of Fe-BTC was prepared via treatment for 30 min in an ultrasonic bath at 22 kHz. Separately, 5 wt % of aqueous EDTANa solution was obtained and stirred with a magnetic stirrer. The Fe-BTC dispersion and EDTANa solution were mixed in the ratio of 1:2.5 by weight. Then, a calculated amount of the Fe-BTC-EDTANa dispersion was added to the ChS/MA solution to obtain ChS/Fe-BTC at a ratio of 1:0.3 by weight, mixed for 10 min using a magnetic stirrer, and then sonicated for 30 min. The Fe-BTC concentration was selected according to our previous study [83]. 

The ChS and ChS/Fe-BTC interlayers on the surface of porous PAN membrane-support were prepared via dead-end ultrafiltration of ChS/MA aqueous solution or ChS/MA/Fe-BTC aqueous dispersion, through PAN membrane support at 3 bar, using an Amicon-type ultrafiltration cell. Two interlayers of the same composition were formed. The filtration time for the first interlayer was 10 min, while for the second, it was 3 min. The first layer of the composite membrane was left for 2 h at room temperature; thereafter, the second layer was deposited. The obtained membranes were dried for 2 h at 110 °C in an oven to allow the crosslinking of ChS by MA [83].

#### 2.2.2. Formation of the Polyamide Selective Layer by Interfacial Polymerization 

First, 0.1 wt % TETA aqueous solution and 0.05 wt % TMC solution in Nefras C2 were used to obtain a polyamide (PA) layer on the surface of dynamic TFC (ChS/PAN) and TFN (ChS-Fe-BTC/PAN) membranes. The PA selective layer was applied using the following technique. First, the membrane was immersed in the TETA solution for 10 s, followed by the removal of excess moisture and drying at room temperature for 10 min. After that, the membranes were immersed in the TMC solution for 10 s and dried for 10 min at room temperature. To remove the monomer residues, the membranes were kept in ethanol for 15 min. The resulting TFC and TFN membranes were dried for 16 h at 50 °C in an oven.

TFC and TFN membranes with a PA selective layer were modified by introducing 0.01–0.05 wt % Fe-BTC into the 0.05 wt % TMC solution in the Nefras C2 during IP. The TMC-Fe-BTC dispersions were treated with ultrasound for 30 min before application in interfacial polymerization. The TFC and TFN membrane abbreviations and preparation conditions are presented in Table 1.

### 2.3. Membrane Characterization

#### 2.3.1. Scanning Electron Microscopy (SEM)

The membrane morphology was studied using the Phenom Pro (Thermo Fisher Scientific, Waltham, MA, USA) and Zeiss Merlin (Carl Zeiss AG, Oberkochen, Germany) scanning electron microscopes. To study the cross-sectional structure, the TFC and TFN membranes were fractured in liquid nitrogen and then sputter-coated with gold to a layer of 1 nm thickness by a vacuum sputter coater DSR (Vaccoat, London, UK).

The composition of the surface of the membrane’s selective layers was studied using a Zeiss Merlin microscope equipped with an energy-dispersive X-ray (EDX) microanalysis instrument (INCA X-Act, Oxford Instruments, Abingdon, Oxfordshire, UK). The accelerating voltage was 15 kV for studying the composition of the membrane surface, and 2 kV for studying the membrane structure. The beam current was 2 nA for studying composition and 100 pA for studying membrane structure. To prevent the accumulation of a charge on the membrane surface, the samples were sputtered with a layer of carbon with a thickness of 15 nm.

#### 2.3.2. Atomic Force Microscopy (AFM)

The topography of the selective layer surface of the TFC and TFN membranes was investigated using an NT-MDT nTegra Maximus atomic force microscope with standard silicon cantilevers, with a stiffness of 15 N·m^−1^ (NT-MDT Spectrum Instruments, Zelenograd, Russia).

#### 2.3.3. Contact Angle Measurement

The water contact angles of the selective layer surface of TFN and TFC membranes were determined by the sessile drop method, using an LK-1 goniometer (Otktrytaya Nauka, Krasnodar, Russia). Measurements were taken 5 s after the formation of a drop on the membrane surface; the measurement error was lower than ± 2⁰.

#### 2.3.4. Average Particle Size

The average particle size of Fe-BTC in 0.05 wt % TMC solution in Nefras C2 was determined using a Zetasizer ZS Nano (Malvern Panalytical, Malvern, UK).

#### 2.3.5. Pervaporation Experiment

The membrane transport properties were studied using the vacuum pervaporation separation of isopropanol/water mixtures with 12 wt %, 20 wt %, and 30 wt % water content, at a feed mixture temperature of 25 °C. The setup for pervaporation is described in detail elsewhere in the literature [84,85,86]. The downstream pressure was less than 0.01 mmHg. The component concentrations in the feed and permeate solutions were determined using a Chromatec Crystal 5000.2 gas chromatograph (Chromatec, Republic of Mari El, Yoshkar-Ola, Russia). The membrane permeation flux (J, g·m^−2^·h^−1^) was calculated according to Equation (1):

(1) $$J=\frac{m}{S\times t},$$

where *m* is the weight of the permeate, g, *S* is the effective membrane area in m^2^, and *t* is the time of measurement, in h.

## 3. Results and Discussion

### 3.1. Structure and Hydrophilic–Hydrophobic Balance of TFC and TFN Membranes with ChS and ChS-Fe-BTC Interlayers

#### 3.1.1. Investigation of the Membrane Structure via SEM and AFM

The ChS or ChS-Fe-BTC interlayer was deposited on the surface of a porous membrane substrate via the dynamic technique (dead-end ultrafiltration of aqueous ChS solution or ChS-Fe-BTC aqueous dispersion, through a porous PAN membrane). The structure, physicochemical properties, and pervaporation performance of ChS/PAN and ChS-Fe-BTC TFC membranes were discussed in detail in [83]. It was found that the introduction of 30 wt % of Fe-BTC with respect to the ChS weight yields an increase in the thickness of the membrane selective layer (from 0.44 to 4.53 µm), along with the roughness (average surface roughness (R_a_) increased from 3.44 to 8.95 nm) and water contact angle (increased from 30 ± 2° to 36 ± 2°) of the selective layer surface. Moreover, both permeation flux and water content in the permeate increased in the process of isopropanol dehydration via pervaporation. Permeation flux increased from 51–203 to 95–494 g·m^−2^·h^−1^ in pervaporation dehydration of isopropanol with a water content of 12–30 wt % in the feed mixture [83]. 

This work deals with the further modification of ChS/PAN and ChS-Fe-BTC/PAN membranes by the formation of polyamide (PA) ultrathin selective layers via IP. Moreover, the TFC interlayered membranes were further modified by the introduction of Fe-BTC into the PA selective layer. The influence of Fe-BTC incorporation into the interlayer and an ultrathin selective layer on the structure and performance of the TFN membrane was thoroughly investigated (membrane preparation conditions and membrane abbreviations are shown in Table 1).

**Figure 1:**
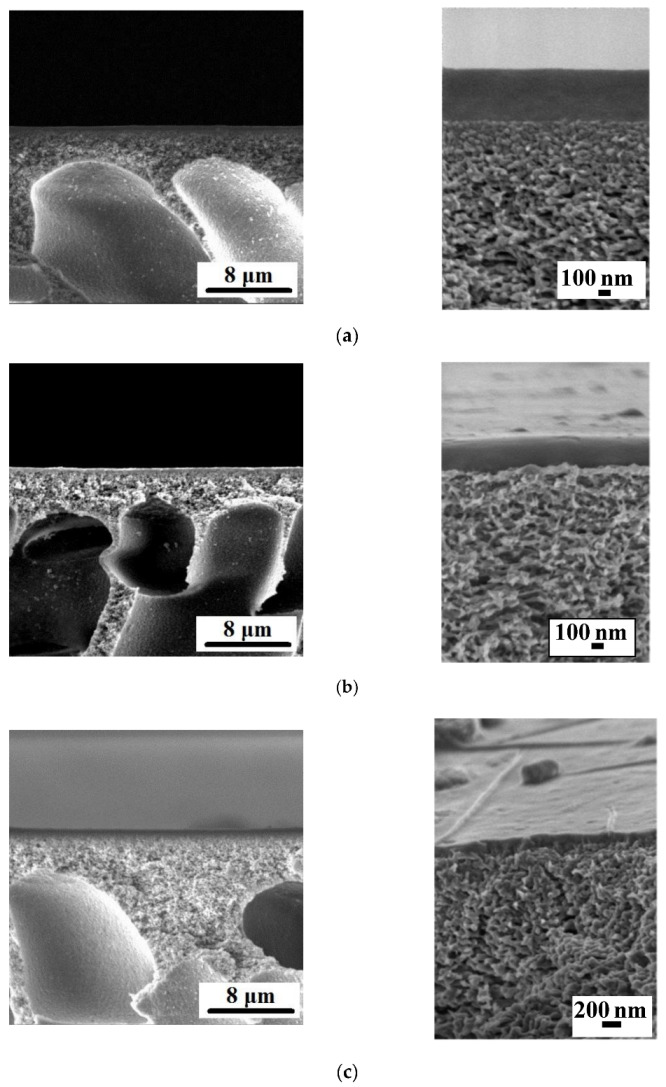

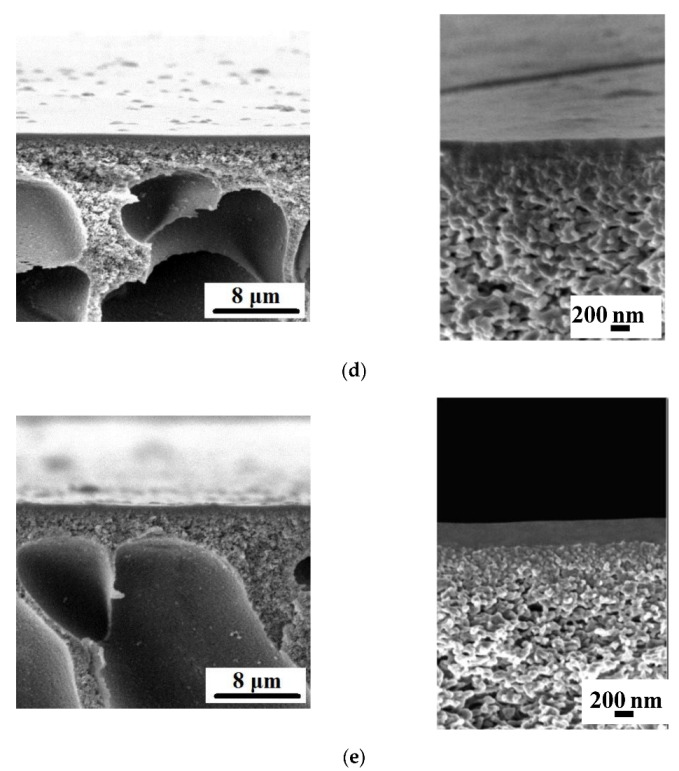
SEM micrographs of the cross-section of the selective layer of TFC and TFN membranes at higher and lower magnification: (**a**) D0; (**b**) D0-IP; (**c**) D0-IP1; (**d**) D0-IP3; (**e**) D0-IP5.

**Figure 2:**
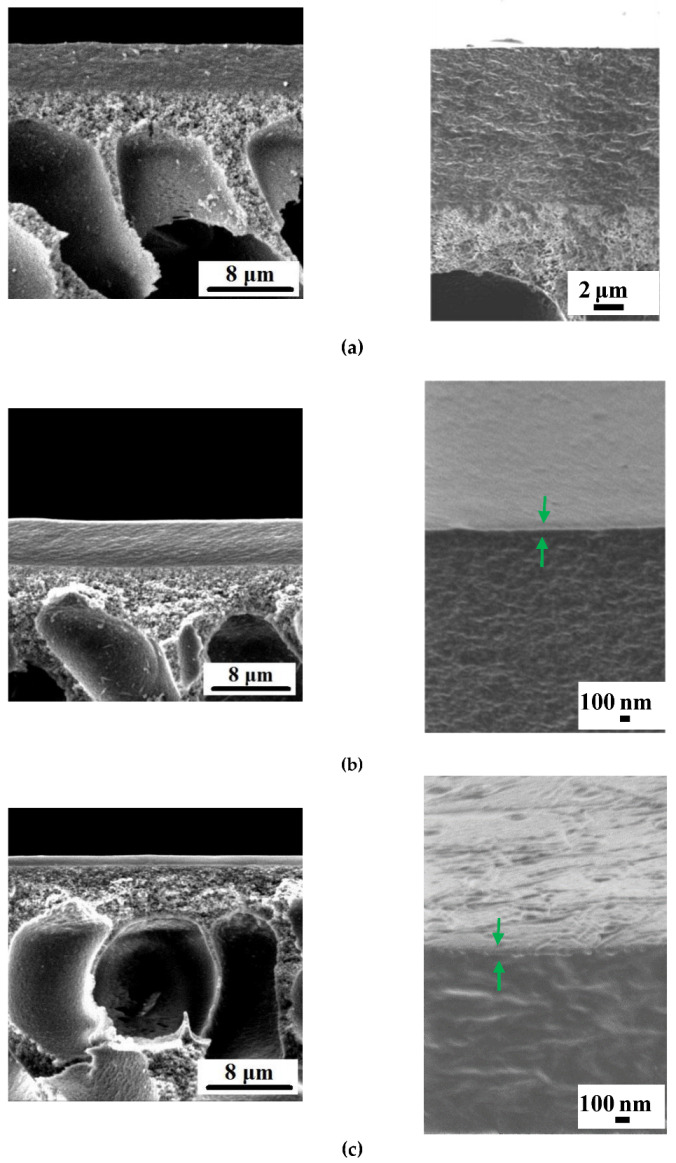

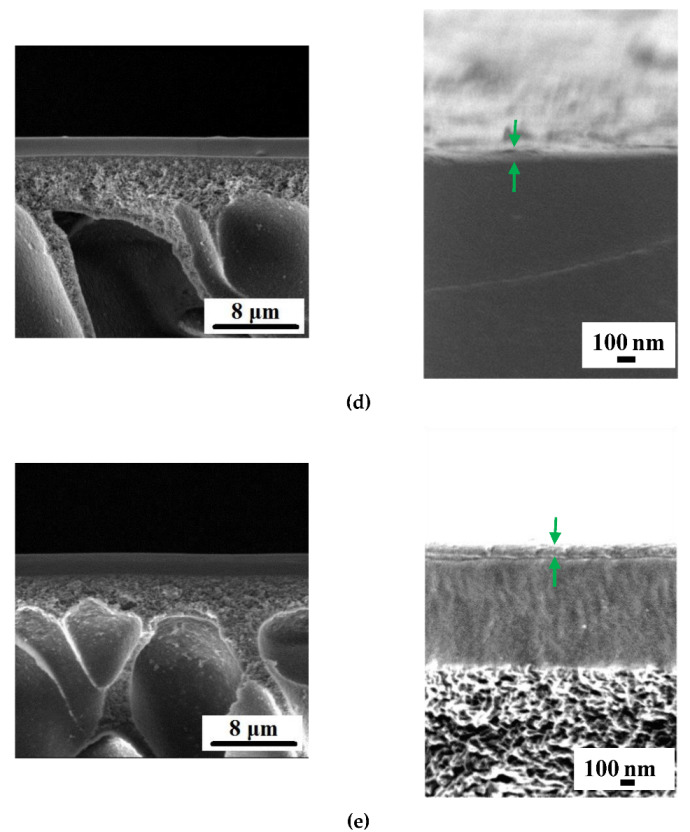
SEM micrographs of the cross-section of the selective layers of TFC and TFN membranes at higher and lower magnifications: (**a**) D30; (**b**) D30-IP; (**c**) D30-IP1; (**d**) D30-IP3; (**e**) D30-IP5. The arrows show the PA selective layer prepared via IP.

**Figure 3:**
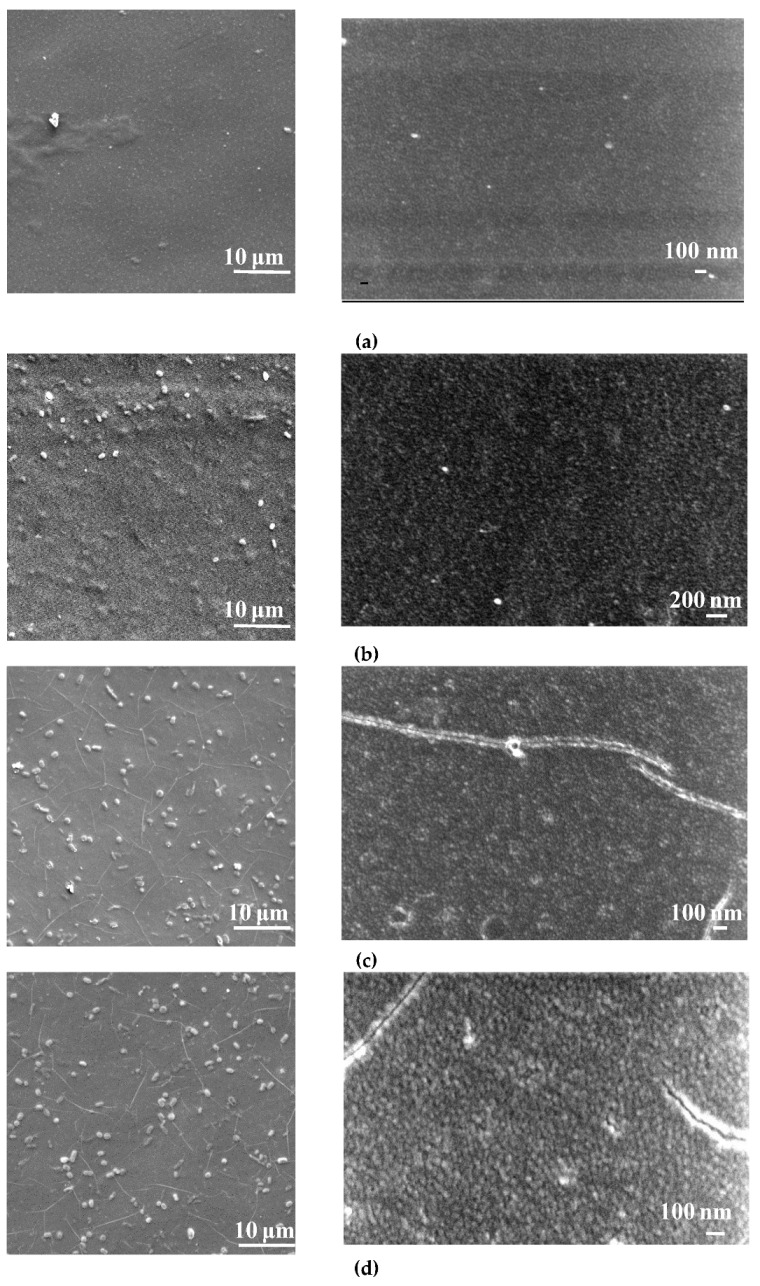

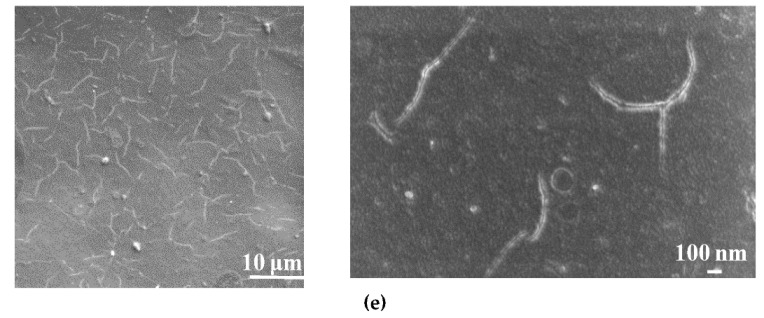
SEM micrographs of the surfaces of the selective layer of the TFC and TFN membranes at lower and higher magnification: (**a**) D0; (**b**) D0-IP; (**c**) D0-IP1; (**d**) D0-IP3; (**e**) D0-IP5.

**Figure 4:**
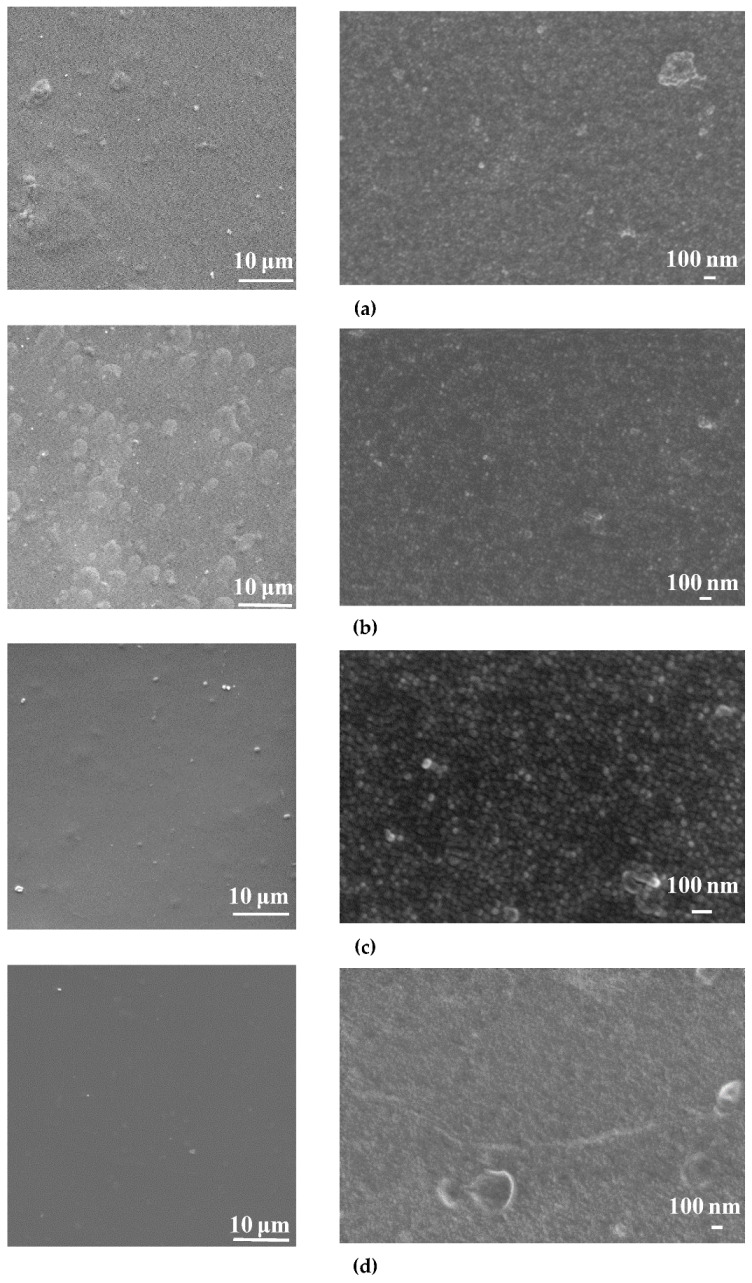

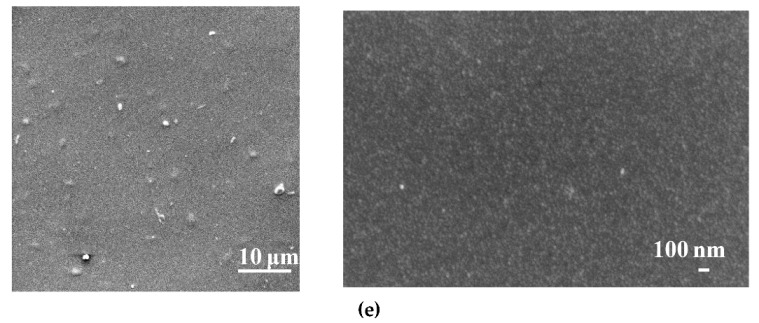
SEM micrographs of the surfaces of the selective layer of TFC and TFN membranes at lower and higher magnification: (**a**) D30; (**b**) D30-IP; (**c**) D30-IP1; (**d**) D30-IP3; (**e**) D30-IP5.

Сross-section morphologies and the selective layer surfaces of the TFC and TFN membranes developed within this study are presented in Figure 1, Figure 2, Figure 3 and Figure 4. The thicknesses of the selective layer of TFC and TFN membranes according to the SEM microphotographs are shown in Table 2.

As was discussed previously, the incorporation of 30 wt % Fe-BTC with respect to ChS results in a substantial increase in the thickness of the membrane selective layer, due to the increase in concentration polarization, the viscosity of the ChS solution, and the additional cross-linking of ChS chains during selective layer formation via a dynamic technique that was reported in our previous study (Figure 1a and Figure 2a; Table 2) [83]. It was found that the formation of the polyamide (PA) ultrathin selective layer by IP yields a slight decrease in the overall thickness of the selective layer of both the ChS/PAN (D0-IP) and ChS-Fe-BTC/PAN (D30-IP) membranes (Figure 1 and Figure 2; Table 2). This slight decrease is due to the swelling of the ChS and ChS/Fe-BTC interlayer in the aqueous solution of TETA, during the formation of an ultrathin selective layer by IP. When the formed hierarchically structured layer is dried, shrinkage occurs due to capillary forces, which lead to a slight decrease in thickness.

It was found that dispersions of 0.01–0.05 wt % of Fe-BTC in 0.05 wt % TMC solution in Nefras C2 are stable for more than 5 days and are characterized by an average particle size of 80–120 nm. It was shown that when 0.01 wt % Fe-BTC was added to the TMC solution in Nefras C2, a further decrease in the overall selective layer thickness was observed for both membranes with ChS and ChS-Fe-BTC interlayers. According to the SEM images of the membrane cross-section, the thickness of PA selective layers for ChS-Fe-BTC interlayered membranes was 80–100 nm (Figure 2). The PA layer on the surface of the ChS interlayered membranes was not clearly seen; therefore, the thickness was not determined (Figure 1). 

However, this decrease is dramatic in the case of the D30-IP1 membrane: the thickness decreased by 80% compared to the D30-IP membrane, decreasing from 4.34 down to 0.87 µm (Figure 1 and Figure 2; Table 2). For D0-IP1, the thickness was found to decrease only by 30% compared to the D0-IP membrane (Figure 1; Table 2). This difference is attributable to the different structure of the interlayer, which influences the process of the PA layer formation by IP [29,30,31,32,33]. When the Fe-BTC concentration increases from 0.01 wt % to 0.03 wt % and 0.05 wt % in the TMC solution, in Nefras C2, the selective layer thickness increases for both the ChS and ChS-Fe-BTC interlayered membranes (Figure 1; Table 2). The thickness of the D0-IP5 membrane even surpasses the thickness of the D0-IP membrane by 75% (0.70 and 0.40 µm, respectively). On the other hand, the overall thickness of the hierarchically structured selective layer for the D30-IP3 and D30-IP5 (1.70 and 1.98 µm) membranes is significantly lower compared to the D30-IP and D30 membranes (4.34 and 4.53 µm) (Figure 1 and Figure 2; Table 2). The dramatic decrease in the overall thickness of hierarchically structured selective layer for membranes with ChS-Fe-BTC interlayer may be due to the increased free volume of the interlayer, which leads to the increased penetration of the TETA aqueous solution inside the interlayer and the high degree of shrinkage of the selective layer after drying. 

**Figure 5:**
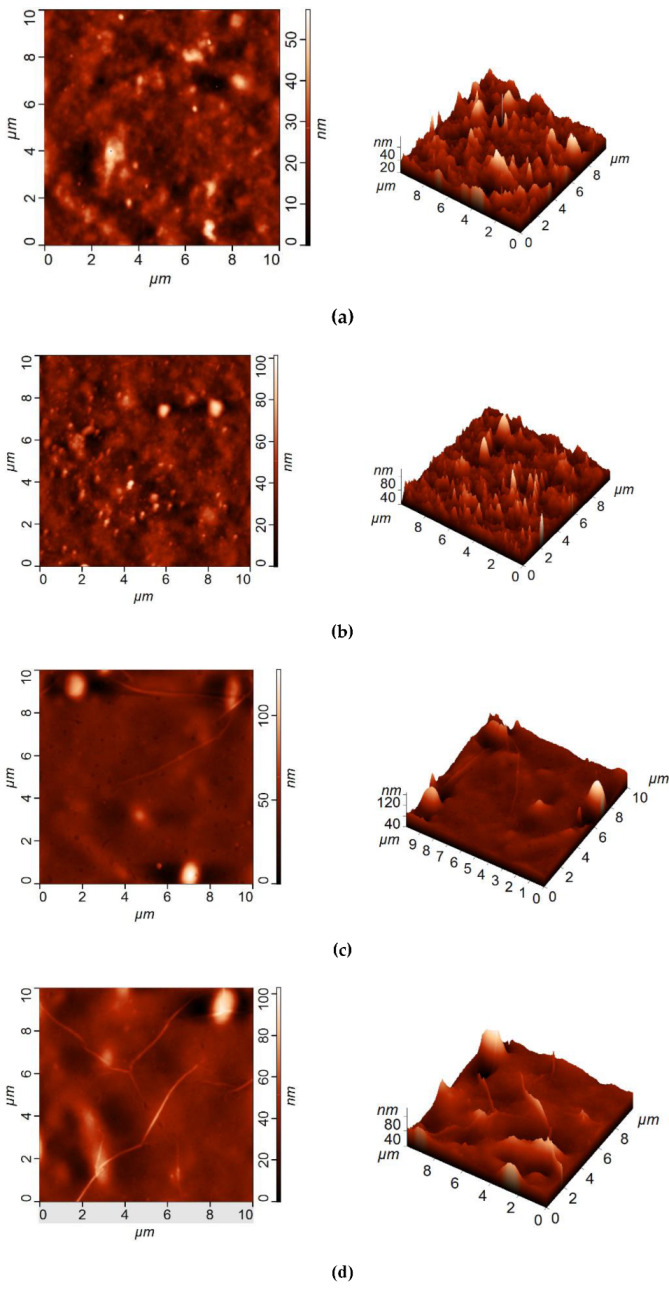

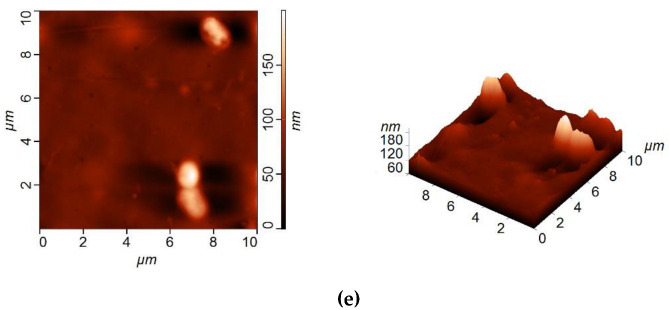
AFM images of the selective layer surface of the TFC and TFN membranes: (**a**) D0; (**b**) D0-IP; (**c**) D0-IP1; (**d**) D0-IP3; (**e**) D0-IP5.

**Figure 6:**
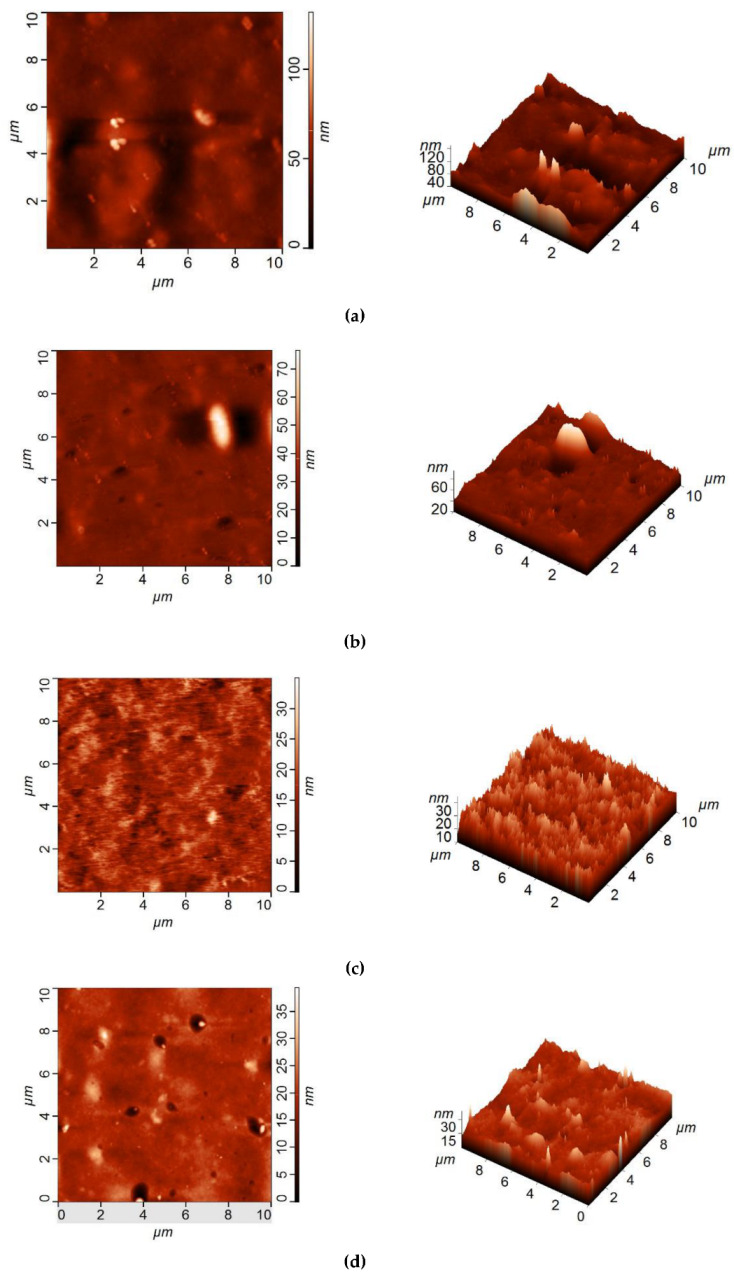

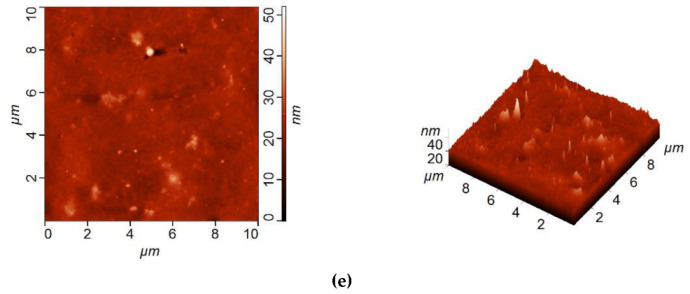
AFM images of the selective layer surface of TFN membranes: (**a**) D30; (**b**) D30-IP; (**c**) D30-IP1; (**d**) D30-IP3; (**e**) D30-IP5.

The surfaces of the selective layers of the membranes were studied using the SEM and AFM techniques (Figure 3, Figure 4, Figure 5 and Figure 6; Table 3). Energy-dispersive X-ray (EDX) microanalysis was used to prove the incorporation of Fe-BTC into the PA selective layer. No Fe atoms were detected on the surface of the selective layer of the D0 and D0-IP membranes. It was found that the surface of the D30-IP5 membrane contained 1.2 at % of Fe and D0-IP5 contains 0.2 at % of Fe. The much higher content of Fe for D30-IP5 compared to the D0-IP5 membrane is attributed to the presence of 30 wt % of Fe in the ChS interlayer.

**Figure 7:**
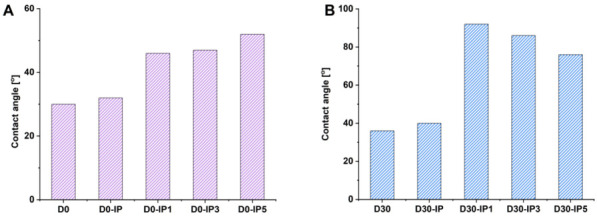
Water contact angles of the developed TFC and TFN membranes with ChS (**A**) and Fe-BTC-ChS (**B**) interlayers.

The surface roughness parameters (“root-mean-square” roughness (R_q_) and average roughness (R_a_)) are presented in Table 3. It was found that the structure and composition of the interlayer (ChS or ChS/Fe-BTC) greatly influence the structure of the ultrathin selective layer formed via the IP technique. It was previously shown that when Fe-BTC is embedded into the ChS interlayer, agglomerates of Fe-BTC appear on the surface of the membrane selective layer (Figure 3 and Figure 4), which leads to a substantial increase in surface roughness (Figure 5 and Figure 6; Table 3) and a slight increase in water contact angle (from 30 ± 2° to 36 ± 2°) (Figure 7) of the selective layer surface [83]. It is commonly known that the contact angle also depends on the surface roughness parameters, along with the chemical nature of the surface [87]. 

It was revealed that the ultrathin selective layer formed by IP on the ChS interlayer (Figure 3b) features polymer/oligomer globules on the surface, unlike the surface of the smoother and more uniform PA layer that formed on the ChS-Fe-BTC interlayer (Figure 4b). The formation of a nodule structure in the PA layer is usually due to rapid and violent amine diffusion and, thus, the formation of large initial polyamide oligomers in the beginning stage of IP. When ChS/PAN or a ChS-Fe-BTC/PAN membrane is immersed in a TETA aqueous solution, the highly hydrophilic cross-linked interlayer swells and the TETA solution penetrates inside the layer. In spite of the hydrophilic and continuous structure of the ChS interlayer, it seems not to be able to efficiently regulate amine diffusion near the interface of the aqueous and organic phase because of the absence of pores, which usually serve as storage reservoirs for amines [29,30,31,32,33]. Instead, the ChS-Fe-BTC interlayer features a less tightly packed structure with high free volume- and size-defined regions of embedded amorpho-crystalline Fe-BTC particles, which serve as a TETA storage reservoir. The TETA molecules are efficiently trapped inside the Fe-BTC structure, also due to the hydrogen bond formation between the hydrogen atoms of amine and the oxygen atoms of 1,3,5-benzenetricarboxylate. From one point of view, it ensures the increased storage of amine molecules near the interface, which is important for the formation of a defect-free PA selective layer, with enhanced rejection [29,30,31,32,33]. On the other hand, this provides a reduced amine desorption rate and slowed amine diffusion, which yields the formation of a smooth and uniform PA layer by IP [29,30,31,32,33]. The results of the investigation of the membrane’s surface by SEM are in good agreement with AFM studies (Figure 5b and Figure 6b; Table 3). It was found that the D0-IP membrane is characterized by much higher surface roughness parameters and the presence of globules, compared to the D30-IP membrane (Figure 5b and Figure 6b; Table 3). Moreover, the creation of an IP layer on the ChS-Fe-BTC interlayer results in decreasing surface roughness parameters compared to the D30 membrane, which is due to the smooth and uniform PA layer formation, unlike the D0-IP membrane, which is characterized by higher roughness compared to the D0 membrane (Figure 5a,b and Figure 6a,b; Table 3). A substantial decrease in the surface roughness of the D30-IP membrane compared to the D30 membrane can be attributed to the filling of valleys and irregularities in the selective layer surface of the D30 membrane with the PA layer. 

When Fe-BTC is added to the TMC solution in Nefras C2, the structure of the membrane surface with the ChS interlayer changes dramatically (Figure 3c–e). The introduction of 0.01 wt % Fe-BTC into the PA selective layer results in the creation of a strand hybrid morphology in the PA selective layer, along with an increase in the number and size of polyamide oligomer nodules. The increase in Fe-BTC concentration up to 0.05 wt % yields an increase in the number of strands and a substantial decrease in the number of polyamide oligomer nodules (Figure 3c–e). The results of the AFM investigations are consistent with the results of SEM studies (Figure 5c–e; Table 3). Large PA globules and long strands are observed on the surface of the D0-IP1, D0-IP2, and D0-IP3 membranes (Figure 3c–e). Surface roughness parameters for the D0-IP1 membrane surface are lower compared to the D0-IP due to the change in topography and the appearance of strands, which are relatively flat, and a decrease in the density of occurrence of the polyamide globules (Table 3). However, AFM studies have revealed that the size of the globules increases with the rise in Fe-BTC concentration in the TMC solution in Nefras C2 (Figure 5b–e). The strand hybrid morphology of the polyamide layer has been observed and discussed in a previous study [88]. In this study, an interlayer on the surface of the ultrafiltration and microfiltration membrane support was formed via the co-deposition of a macrocycle polyphenol molecule (Noria) and polyethyleneimine (PEI). Then, a TFC nanofiltration membrane with an ultrathin selective layer was formed via the IP of piperazine (PIP) and TMC [88]. It was found that the strand morphology of the PA selective layer surface was due to the host–guest interaction between PIP and the anchored Noria. Subsequently, the as-formed Noria–PIP–TMC complex served as the original location for the IP reaction, during which PIP erupted from the support surface pores and paired with the TMC in hexane to participate in the growth of strands [88].

In the case of membranes with a ChS interlayer, the fast and vigorous diffusion of amine to the TMC solution in Nefras C2 at the aqueous-organic interface yielded its entrapment in the Fe-BTC cavities in the TMC solution in Nefras C2. These Fe-BTC cavities, with trapped TETA molecules, can serve as the original location of the IP reaction, the point from which the PA chains start to grow. It leads to the formation of the hybrid strand morphology of the PA layer, similar to the case reported in [88]. The increase in the number of strands and the decrease in the number of oligomer globules on the membrane’s surface at the highest studied concentration of Fe-BTC (D0-IP5 membrane) supports this assumption (Figure 3e). It can be concluded that the incorporation of Fe-BTC in the TMC solution slows down the rate of IP reaction, due to the entrapment of TETA molecules inside the Fe-BTC cavities. It can be concluded that the incorporation of Fe-BTC in the TMC solution slows down the rate of IP reaction, due to the entrapment of TETA molecules inside the Fe-BTC cavities. Moreover, the formation of the hydrogen bond of TETA and 1,3,5-benzenetricarboxylate may occur with TMC. It hinders the formation of oligomer globules on the membrane surface and contributes to the increase in the size of globules with the increase in Fe-BTC concentrations in the PA layer (Figure 3e and Figure 5e) due to the decrease in the IP rate.

When 0.01–0.03 wt % Fe-BTC was added to the TMC solution in Nefras C2 for a ChS-Fe-BTC interlayered membrane, a smooth surface to the PA selective layer is formed (Figure 4c–e) and the surface roughness parameters decreased, even compared to the D30-IP membrane (Figure 6b–d; Table 3). Such a smooth and uniform surface is due to the decrease in the rate of amine desorption and diffusion due to its entrapment in Fe-BTC cavities, both in the interlayer and in the TMC solution near the reaction interface, and the decrease in the reaction rate. It is worth noting that the Fe-BTC in ChS is dispersed using the excess of EDTANa, which possesses two free carboxyl groups that are not substituted by sodium cations. EDTANa could (i) react with TETA while the TETA impregnated the ChS-Fe-BTC interlayer and at the reaction interface, substituting TMC, and (2) diffuse into the TMC solution in Nefras C2 and facilitate the better dispersion of Fe-BTC in the PA layer. Overall, this contributes to the formation of a smooth and uniform selective PA layer. The formation of a hybrid strand morphology and oligomer globules did not occur in the D30-IP1, D30-IP3, and D30-IP5 membranes since the fast and vigorous diffusion of TETA into the water–organic reaction interface was hindered by the entrapment of TETA in the Fe-BTC cavities of the ChS-Fe-BTC interlayer when it was impregnated with the TETA aqueous solution prior to IP reaction.

When the Fe-BTC concentration in the TMC solution was increased up to 0.05 wt %, some nodules were observed on the surface of the selective layer (Figure 4e); however, this did not increase the membrane’s surface roughness (Figure 6e; Table 3). This may have been due to aggregated Fe-BTC nanoparticles or Fe-BTC-PA hybrid nanoparticles.

The results obtained demonstrate the crucial role of the structure and chemical nature of the interlayer on the process of PA layer formation via IP. It can be concluded that the simultaneous incorporation of Fe-BTC in the ChS interlayer and TMC solution during IP can effectively regulate the rate of amine diffusion and result in the formation of a uniform and smooth PA selective layer.

#### 3.1.2. Studies of the Water Contact Angle of TFC and TFN Membranes

The water contact angle, along with the structure and topography, is an important characteristic of the membrane selective layer in terms of pervaporation, which influences membrane performance. Figure 7 presents the water contact angles of the surface of the membrane selective layer for the developed TFC and TFN membranes.

As was reported in our previous work, the incorporation of 30 wt % Fe-BTC, according to ChS weight, resulted in the increase in the water contact angle of the selective layer surface from 30 ± 2° to 36 ± 2°, due to the presence of Fe-BTC hydrophobic units in the selective layer and an increase in the degree of surface roughness (Figure 5a, Figure 6a and Figure 7; Table 3) [83]. When the PA selective layer is formed on the surface of D0 and D30 membranes via IP, the water contact angle slightly increases, up to 32 ± 2° and 40 ± 2°, respectively. However, the degree of surface roughness increases for the D0-IP membrane and decreases for the D30-IP membrane, compared to the D0 and D30 membranes, respectively (Table 3). Therefore, the increase in contact angle for the D30-IP membrane combines with the decrease in surface roughness compared to the D30 membrane. This opposite trend can be explained by the probably different composition of the PA ultrathin selective layer due to the influence of the interlayer’s structure and composition on the IP reaction. It may be suggested that in the case of TFN membranes with an Fe-BTC embedded interlayer, the rate of TETA desorption and diffusion to the reaction interface is decreased, while a PA with a lower content of hydrophilic TETA molecules is formed during IP. It is known that an increase in the number of acyl fragments, compared to the amount of a multifunctional amine during IP, leads to the hydrophobization of the selective layer [89].

It was found that when Fe-BTC was further introduced into the PA selective layer, the water contact angle increased for both the ChS and ChS-Fe-BTC interlayered membranes, in spite of the different trends in the change in surface roughness parameters (Figure 7; Table 3). It was found that in the ChS interlayered membranes, D0-IP1 and D0-IP3, the water contact angles were in the range of 46–47 ± 2° (Figure 7A). When the Fe-BTC concentration increased up to 0.05 wt %, the contact angle increased up to 52 ± 2° (Figure 7A). The main reasons for the increase in water contact angle were the incorporation of Fe-BTC hydrophobic units into the PA selective layer and the significantly increased surface roughness parameters (Table 3). 

It was revealed that for the TFN membranes with an Fe-BTC-ChS interlayer, the water contact angle of the PA/Fe-BTC selective layer increased up to 92 ± 2°, when 0.01 wt % of Fe-BTC was added to the PA layer, and then decreased down to 86 ± 2° for the D30-IP3 membrane and to 76 ± 2° for the D30-IP5 membrane (Figure 7B). The enhanced hydrophobicity of the selective layer surface in the ChS-Fe-BTC interlayered membranes (D30-IP1, D30-IP3, and D30-IP5) compared to the ChS interlayered membranes (D0-IP1, D0-IP3, and D0-IP5) was due to the different structure and chemical composition of the PA layer. The Fe-BTC size-defined structure in the interlayer and PA selective layer facilitated the increased amine storage and entrapment, which led to a decrease in the diffusion rate and, hence, a decrease in the number of active TETA molecules taking part in the reaction with TMC. This results in the formation of a PA layer enriched with hydrophobic acyl chloride units. A decrease in the water contact angle with an increase in Fe-BTC concentration in the TMC solution in Nefras C2 during IP can be attributed to a decrease in the membrane’s surface roughness (Figure 7B; Table 3). 

### 3.2. Effects of Fe-BTC Concentration in the Interlayer and the PA Selective Layer on the Pervaporation Performance of TFC and TFN Membranes

**Figure 8:**
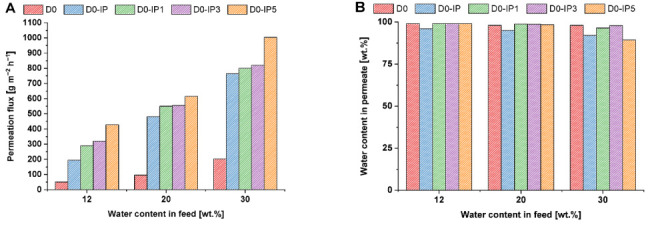
Pervaporation performance of the TFC and TFN membranes with the ChS interlayer. (**A**): permeation flux; (**B**): water content in the permeate.

**Figure 9:**
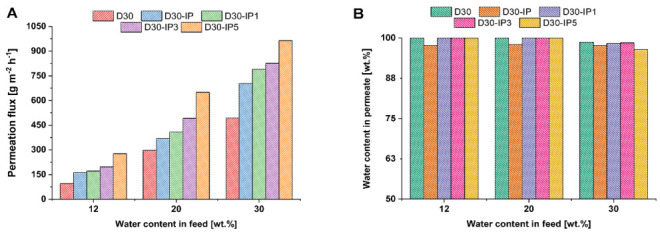
Pervaporation performance of TFC and TFN membranes with ChS-Fe-BTC interlayer: (**A**)—permeation flux; (**B**)—water content in permeate.

The transport properties of the developed hierarchically structured TFC and TFN membranes were studied in the pervaporation of an isopropanol/water mixture with a water content of 12 wt %, 20 wt %, and 30 wt %. This dependence on the permeation flux and water content in the permeate on the water content in the feed for the ChS and ChS-Fe-BTC interlayered TFC and TFN membranes is presented in Figure 8 and Figure 9.

It was shown that when 30 wt % Fe-BTC was embedded into the ChS selective layer, permeation flux increased from 51–203 to 95–494 g·m^−2^·h^−1^, while the water content in the permeate increased from 98–99 wt % up to 98.70–99.99 wt % in the pervaporation dehydration of isopropanol, with a water content of 12–30 wt % in the feed solution (Figure 8 and Figure 9) [83]. It was found that an increase in water content in the feed results in a significant increase in the permeation flux for both the D0 and D30 membranes, which can be attributed to the swelling of the hydrophilic selective layer. However, it was revealed that the D30 membrane features a higher water content in the permeate, which can be assigned to the additional cross-linking of the selective layer (Figure 8B and Figure 9B). This is due to hydrogen bond formation between the carboxylate groups of Fe-BTC and the hydroxyl groups of ChS, along with donor-acceptor bond formation between the free orbitals of Fe atoms and the lone pairs of electrons of the oxygen atoms in the carboxyl and hydroxyl group in ChS [83]. It was found that the formation of a PA ultrathin selective layer via IP yields a substantial increase in permeation flux and a decrease in selectivity for both ChS and ChS-Fe-BTC interlayered membranes (Figure 8 and Figure 9). It was found that the permeation flux of the D0-IP membrane is 195 g·m^−2^·h^−1^ at 12 wt % water content in the feed, 482 g·m^−2^·h^−1^ at 20 wt %, and 765 g·m^−2^·h^−1^ at 30 wt % (Figure 8A). The corresponding permeation fluxes for the D0 membrane are 51, 96, and 203 g·m^−2^·h^−1^. However, the formation of the PA selective layer via IP on the surface of the D0 membrane leads to a decrease in membrane selectivity when the water content in the feed increases due to swelling: the water content in the permeate is 96 wt % with 12 wt % water in the feed, at 95 wt % with 20 wt % water in the feed, and at 92 wt % with 30 wt % water in the feed, which is lower compared to the performance of the D0 membrane (Figure 8B). A similar trend was observed in our previous study, when a PA layer was formed on a chitosan interlayer [51]. This is attributed to the disruption of the ChS hydrogen bond system when the PA layer is formed. In spite of the cross-linking by MA, ChS is able to swell in an aqueous TETA solution, and the amine molecules penetrate between the ChS chains. When impregnated with TETA, the ChS layer makes contact with the TMC solution in Nefras C2, and the following phenomena may occur: (1) the TETA reacts with the TMC and moves the ChS chains apart by the growing PA chains, disrupting the dense packing of the ChS chains in the interlayer; (2) the hydroxyl groups of ChS react with TMC instead of with the TETA and, as a result, the growing PA chains break. Moreover, the disruption of the ChS hydrogen bond system and a decrease in the packing density in the cross-linked ChS layer will occur. Hence, the PA does not then form a dense, defect-free layer. The polyamide partially breaks off and partially intertwines with the ChS, which leads to an increase in the permeation flux and a decrease in selectivity. The increase in permeation flux is also due to the increase in surface roughness of the selective layer surface when the PA layer is formed, which increases the sorption area for penetrants (Table 3).

The increase in permeation flux and the decrease in water content in the permeate were similarly observed for the D30-IP membrane, compared to the transport properties of the D30 membrane (Figure 9).

However, the permeation flux of the D30-IP membrane was slightly lower, at 12–30 wt % water content in the feed (162 g·m^−2^·h^−1^ at 12 wt % water content in the feed, 369 g·m^−2^·h^−1^ at 20 wt %, and 704 g·m^−2^·h^−1^ at 30 wt % of water in the feed) compared to the performance of the D0-IP membrane. However, the selectivity of the D30-IP membrane was higher (97.7–98 wt % water content in the permeate) compared to the selectivity of the D0-IP membrane (92–96 wt %) (Figure 8B and Figure 9B). A lower permeation flux and higher selectivity of the D30-IP membrane compared to the D0-IP membrane was due to the higher thickness of the hierarchically structured selective layer of the D30-IP membrane and the smoother and more uniform PA layer (Figure 1, Figure 2, Figure 3 and Figure 4; Table 2). Thus, the incorporation of Fe-BTC in the ChS interlayer slightly decreased the permeation flux and increased the membrane selectivity and stability in diluted feed mixtures (Figure 8 and Figure 9).

It was found that the introduction of 0.01–0.05 wt % of Fe-BTC into the PA layer that was formed via IP for ChS and Fe-BTC/ChS interlayered membranes yielded a substantial increase in permeation flux compared to the reference for D0-IP, D0, D30-IP, and D30 membranes at all studied feed mixtures (Figure 8A and Figure 9A). This is due to the increase in the PA layer’s free volume and the decrease in the PA chain-packing density when microporous particles of Fe-BTC are embedded in the PA layer.

It was found that the flux of D0-IP1, D0-IP3, and D0-IP5 membranes was higher compared to the D30-IP, D30-IP3, and D30-IP5 membranes (Figure 8A and Figure 9A). It is attributed to the following reasons: the lower thickness of hierarchically structured selective layers, higher surface roughness, and higher hydrophilicity of ChS interlayered TFN membranes compared to the ChS-Fe-BTC interlayered membranes. It was revealed that a less uniform and rougher PA selective layer with oligomer globules and strand hybrid morphology is formed for TFN membranes with ChS interlayer (Figure 3, Figure 4, Figure 5 and Figure 6; Table 3). It is known that higher surface roughness and higher hydrophilicity facilitate the increase in the sorption of feed solution components, which increases the permeation flux in pervaporation. 

It was found that membrane selectivity toward water increased when Fe-BTC was embedded into the PA selective layer, which was expressed in the increase in water content in the permeate (Figure 8B and Figure 9B). This can be assigned to the rise in the difference in the diffusion rates of the molecules of various sizes through the PA selective layer, due to the increase in the length of the diffusion path and the presence of regions with size-defined parameters. Moreover, additional cross-linking and stabilization of the PA structure occurred with the introduction of Fe-BTC in the selective layer, due to the formation of donor-acceptor bonds of the iron atoms of Fe-BTC and the unreacted acyl chloride groups of the formed PA. Additional cross-linking may also have been due to the formation of hydrogen bonds between the carboxylate groups in the structure of Fe-BTC and unreacted amine groups of PA.

Moreover, it was observed that the ChS-Fe-BTC interlayered membranes, D30-IP, D30-IP3, and D30-IP5, demonstrated higher selectivity (higher water content in the permeate) compared to the ChS interlayered membranes, D0-IP1, D0-IP3, and D0-IP5. This is due to the formation of a smoother, more uniform, and defect-free PA layer, which can be attributed to the slowing-down of the rate of TETA diffusion to the IP reaction interface since it is entrapped in the Fe-BTC microporous structure (Figure 3 and Figure 4). Moreover, the ChS-Fe-BTC interlayer contains EDTANa, which can diffuse into the organic phase and facilitate the better dispersion of Fe-BTC particles. This can prevent the formation of non-selective voids and defects at the Fe-BTC/PA interface in the selective layer, which leads to higher membrane selectivity.

It was found that when the Fe-BTC concentration in the TMC Nefras C2 solution increased from 0.01 up to 0.05 wt %, the permeation flux increased for both the ChS and ChS-FeBTC interlayered membranes (Figure 8A and Figure 9A). The highest permeation flux is achieved for the D0-IP5 membrane (428–1004 g·m^−2^·h^−1^ at 12–30 wt % water content in the feed solution) and for the D30-IP5 membrane (277–964 g·m^−2^·h^−1^ at 12–30 wt % water content in the feed solution). Enhanced permeation flux with the increase in Fe-BTC concentration in the organic phase during the PA selective layer formation via IP is due to the introduction of porous Fe-BTC particles with a very high specific surface area and microporous structure, which decreases the packing density of PA chains.

It is worth noting that an increase in Fe-BTC concentration yields a significant decrease in water content in the permeate for ChS-interlayered membranes, down to 89.3 wt % at 30 wt % water content in the feed solution for the D0-IP5 membrane. However, for ChS-Fe-BTC interlayered membranes, the water content in the permeate only slightly decreased at 30 wt % of water content in the feed mixture for the D30-IP1 and D30-IP3 membranes, down to 98.3–98.5 wt %. A more pronounced decrease, down to 96.4 wt % of water content in the permeate, was observed for the D30-IP5 membrane. A decrease in membrane selectivity toward water with an increase in Fe-BTC concentration in the organic phase was due to the lower degree of Fe-BTC dispersion at higher concentrations. 

Thus, better transport properties, in terms of the combination of high permeation flux, high selectivity toward water, and stability in the diluted water/isopropanol feed mixture yielded a D30-IP3 membrane with a permeation flux of 197–826 g·m^−2^·h^−1^ and a water content in the permeate of 98.50–99.99 wt % at 12–30 wt % of water content in the feed solution. 

A comparison of the developed D30-IP3 membrane with the performance of the MOF-based mixed matrix membranes, as reported in the literature, revealed that it demonstrated the highest permeation flux and separation factor when the water content in isopropanol/water feed mixture was 10–20 wt % (Table 4). When the water content in the feed was 30 wt %, D30-IP3 featured the highest permeation flux compared to the other reported MOF-based mixed matrix membranes with a relatively high separation factor, excluding the sodium alginate membrane with the addition of 15 wt % UiO-66 [90] (Table 4). 

## 4. Conclusions

It was found that the structure of the interlayer has a crucial effect on the formation of a polyamide selective layer via interfacial polymerization. The incorporation of a metal–organic framework of Fe-BTC into the chitosan succinate interlayer and polyamide selective layer can effectively regulate the interfacial polymerization reaction via the entrapment of amines and by slowing down its diffusion rate to the reaction interface. This enables the formation of a smoother, more uniform, and defect-free selective layer providing high selectivity, high permeation flux, and high stability toward swelling in the pervaporation of diluted isopropanol/water feed mixtures in thin-film nanocomposite membranes. The developed TFN membranes, with a hierarchically structured selective layer embedded with the Fe-BTC in both the interlayer and polyamide selective layer, can be used for the dehydration of alcohols and organic solvents via pervaporation.

## Figures and Tables

**Table 1:** TFC and TFN membrane abbreviations and preparation conditions (constant parameters: 1 wt % ChS, 15 wt % MA with respect to the ChS weight in aqueous solution).

Abbreviation	Fe-BTC Concentration in ChS Solution (wt % with Respect to ChS Weight)	IP Layer	Fe-BTC Concentration in TMC/Nefras C2 Solution (wt %)
D0	0	-	0
D0-IP		+	0
D0-IP1			0.01
D0-IP3			0.03
D0-IP5			0.05
D30	30	-	0
D30-IP		+	0
D30-IP1			0.01
D30-IP3			0.03
D30-IP5			0.05

“+”—presence of PA layer on the membrane surface prepared via IP; “-”—absence of PA layer on the membrane surface.

**Table 2:** Thickness of the selective layer of TFN membranes.

Membrane Abbreviation	Selective Layer Thickness (µm)
D0	0.44
D0-IP	0.40
D0-IP1	0.28
D0-IP3	0.48
D0-IP5	0.70
D30	4.53
D30-IP	4.34
D30-IP1	0.87
D30-IP3	1.70
D30-IP5	1.98

**Table 3:** Roughness parameters of the selective layer surface of the TFC and TFN membranes.

Membrane Abbreviation	Roughness Parameters	
	R_a_ (nm)	R_q_ (nm)
D0	4.61	6.07
D0-IP	7.17	9.80
D0-IP1	5.71	9.98
D0-IP3	6.61	10.01
D0-IP5	10.75	20.14
D30	7.95	11.74
D30-IP	3.07	6.04
D30-IP1	2.54	3.26
D30-IP3	1.93	2.85
D30-IP5	1.65	2.37

**Table 4:** Comparison of the transport properties of the developed TFN membranes for isopropanol dehydration by pervaporation.

Membranes	Thickness (µm)	Water Content in Feed (wt %)	Temperature (°C)	Permeation Flux (g·m^−2^·h^−1^)	Water Content in Permeate	References
Succinate chitosan/Fe-BTC (40 wt %) (TFN)	4.65	12	25	99	73,326	[83]
		20		296	34,997	
		30		499	23,331	
Succinate chitosan/Fe-BTC (5 wt %) (TFN)	0.43	12	25	180	73,326	[83]
		20		405	34,997	
		30		701	23,331	
Sodium alginate + UiO-66 (15 wt %)/CaCl_2_ (dense)	25	30	22	892	23,000	[90]
Sodium alginate + UiO-66 (15%)/PAN/CaCl_2_ (TFN)	0.7	30	22	872	23,000	[90]
6FDA-HAB/DABA polyimide+ UiO-66 (30%) (dense)	30	15	60	148	~5600	[91]
Polybenzimidazole + ZIF-8 (33.7 wt %) (dense)	50 ± 15	15	60	103	1686	[92]
Polyimide P84 + ZIF-90 (30 wt %) (dense)	~24	15	60	114	385	[93]
Chitosan + ZIF-8 (5 wt %) (dense)	~33	15	60	410	7,236	[94]
PVA/PEG-g-ZIF-8 (15 wt %) (TFN)	1.5 ± 0.3	12	25	91	7,326	[95]
Polyimide/UiO-66-NH_2_ (10 wt %) (dense)	34	15	60	~83	34,997	[96]
Polyimide/UiO-66-NH_2_ (20 wt %) (dense)	47	15	60	~77	34,997	[96]
Polyimide/UiO-66-NH_2_ (30 wt %) (dense)	19	15	60	~216	34,997	[96]
PVA + ZIF-8 (5 wt %) (dense)	70	10	30	868	132	[97]
D30-IP3 (TFN)	1.70	12	25	197	73,326	This work
		20		492	39,996	
		30		826	153.2	

## Data Availability

The data presented in this study are available on request from the corresponding author. The data are not publicly available due to being a part of ongoing research.
